# Relationship between bisphenol A, bisphenol S, and bisphenol F and serum uric acid concentrations among school-aged children

**DOI:** 10.1371/journal.pone.0268503

**Published:** 2022-06-16

**Authors:** Yun Jeong Lee, Youn-Hee Lim, Choong Ho Shin, Bung-Nyun Kim, Johanna Inhyang Kim, Yun-Chul Hong, Yong Min Cho, Young Ah Lee

**Affiliations:** 1 Department of Pediatrics, Seoul National University Children’s Hospital, Seoul, Republic of Korea; 2 Department of Pediatrics, Seoul National University College of Medicine, Seoul, Republic of Korea; 3 Section of Environmental Health, Department of Public Health, University of Copenhagen, Copenhagen, Denmark; 4 Institute of Environmental Medicine, Seoul National University Medical Research Center, Seoul, Republic of Korea; 5 Environmental Health Center, Seoul National University College of Medicine, Seoul, Republic of Korea; 6 Division of Children and Adolescent Psychiatry, Department of Psychiatry, Seoul National University Hospital, Seoul, Republic of Korea; 7 Department of Psychiatry, Hanyang University Medical Center, Seoul, Republic of Korea; 8 Department of Preventive Medicine, Seoul National University College of Medicine, Seoul, Republic of Korea; 9 Department of Nano Chemical and Biological Engineering, SeoKyeong University, Seoul, Republic of Korea; Universita degli Studi del Molise, ITALY

## Abstract

**Background:**

Hyperuricemia has a suspected relationship with hypertension, metabolic syndrome, kidney disease, and cardiovascular disease. Endocrine disruptors may affect uric acid metabolism; however, few epidemiologic studies have been performed in children regarding newly developed bisphenol A (BPA) substitutes. We evaluated the associations between BPA, bisphenol S (BPS), and bisphenol F (BPF) exposure and serum uric acid concentrations in 6-year-old Korean children.

**Methods:**

From the Environment and Development of Children cohort study, six-year-old children (N = 489; 251 boys) who underwent an examination during 2015–2017 were included. Anthropometry, questionnaires, and biological samples were evaluated. BPA, BPS, and BPF levels were measured from spot urine samples, and log-transformed or categorized into groups for analysis. We constructed linear regression models adjusting for age, sex, urinary creatinine levels, body mass index z-scores, and estimated glomerular filtration rates.

**Results:**

Mean serum uric level was 4.2 mg dL^-1^ (0.8 SD) without sex-differences. Among the three bisphenols, higher BPS exposure was associated with increased serum uric acid concentrations (*P*-value for trend = 0.002). When BPS levels were categorized into three groups (non-detection < 0.02 μg L^-1^ vs. medium BPS; 0.02–0.05 μg L^-1^ vs. high BPS ≥ 0.05 μg L^-1^), the high BPS group showed higher serum uric acid concentrations (by 0.26 mg dL^-1^, *P* = 0.003) than the non-detection group after adjusting for covariates, which was significant in boys but not girls.

**Discussions:**

Urinary BPS levels was positively associated with serum uric acid concentrations in 6-year-old children, and the association was more pronounced in boys. Considering the increasing use of BPS and concerning effect of hyperuricemia on health outcomes, their positive relationship should be investigated further.

## Introduction

Uric acid is the end product of an exogenous pool of purines derived from diet and animal proteins, as well as endogenous purine metabolism from the liver, intestines, muscles, kidneys, and vascular endothelium [1]. Although extracellular uric acid acts as a strong antioxidant, intracellular uric acid acts as a pro-oxidant when it stimulates NADPH oxidase and increases oxidative stress [2]. Hyperuricemia has been identified as a cause of gout and nephrolithiasis, and its relationship with hypertension, metabolic syndrome, kidney disease, and cardiovascular disease in adult [3] and pediatric populations has been proposed [4–7].

The prevalence of hyperuricemia has increased in all ages over the past few decades [8], and its prevalence in pediatric populations is reported as 9.4%–34% depending on patient age, sex, ethnicity, and obesity [4–6, 9]. A nationwide study from 2019 has reported the prevalence of hyperuricemia as 9.4% among Korean children and adolescents aged 10–18 years [9]. A Western lifestyle, increased fructose consumption, and increasing prevalence of obesity are largely to blame for the increasing trend of hyperuricemia in this population [8]. Meanwhile, the relationship between environmental chemicals and cardiovascular and metabolic disease has been suggested to be mediated by hyperuricemia in some studies [2, 10].

Bisphenol A (BPA) is a synthetic compound with two functional phenol groups that is used in polycarbonate plastic bottles and toys, epoxy resins, thermal papers, and the lining of food cans [11]. As with adults, children can be exposed to BPA through both dietary and non-dietary sources [12]. BPA has been linked to obesity, diabetes, metabolic syndrome, and thyroid dysfunction in epidemiologic studies [11, 13]. The widespread use of this compound, and the concerning health effects associated with it, have led to the global regulation of BPA by authorities [14–18]. As a result, so-called “BPA-free” substitutes such as bisphenol S (BPS) and bisphenol F (BPF) have become increasingly common [19–21]. However, BPS and BPF may be not safe since their endocrine-disrupting effects have been demonstrated in experimental studies [20]. Nonetheless, data on BPS and BPF exposure and consequent health effects are lacking in pediatric populations [22, 23].

In this study, we investigated the relationship of urinary BPA, BPS, and BPF levels with serum uric acid concentrations in 6-year-old Korean children.

## Material and methods

### Study population

We used data from the Environment and Development of Children (EDC) cohort study, a prospective cohort study to investigate the influence of early-life environmental exposure on physical and neurobehavioral development as previously described in prior research [24]. In brief, the EDC cohort study included children whose mothers participated the Congenital Anomaly Study (CAS), a birth cohort study investigating the association between prenatal environmental exposure and occurrence of congenital anomalies. The CAS study included 11,085 mothers from the metropolitan areas of Seoul and Incheon, in the Republic of Korea. After finishing the CAS study in 2011, we randomly selected and contacted 2,085 mothers between 2012–2015, and a total of 726 mother-child pairs were enrolled in the EDC study. All participants visited Seoul National University Children’s Hospital to undergo a physical examination and laboratory evaluation at the 2-year interval. Among the 574 children who visited the hospital at 6 years of age between 2015–2017, 489 were included in this study after excluding those without urinary BPS and BPF data (n = 79), or without blood samples (n = 6). Written informed consent was obtained from all participants and parents in line with the requirements of the Institutional Review Board of the Seoul National University College of Medicine (IRB no. 1201-010-392). It was confirmed that all procedures were performed in accordance with the relevant guidelines and regulations.

### Anthropometric assessments and questionnaires

Height (cm) was measured using a stadiometer, and weight was measured using a digital scale. Body mass index (BMI) was calculated as weight height^-2^ (kg m^-2^). The height, weight, and BMI z-scores were determined according to 2007 Korean National Growth Charts. Overweight and obesity were defined as a BMI in the 85^th^ to 95^th^ percentile, and above the 95^th^ percentile, respectively [25].

A structured questionnaire was used to collect data on socioeconomic status (monthly household income), environmental tobacco smoke exposure, and physical activity levels (S1 Appendix) [26]. Dietary information was collected from the participants’ mothers using a food frequency questionnaire that evaluated food consumption frequency and portion sizes (S2 Appendix). Daily energy and dietary animal protein intakes were calculated using the Computer Aided Nutritional Analysis Program 4.0 for Professionals (Korean Society of Nutrition, Seoul, Republic of Korea) [27]. Sugar-sweetened beverages (SSBs) included all types of carbonated beverages, soft drinks, sports and energy drinks, fruit juice, flavored milk, yogurt drinks, and sweetened tea or coffee drinks. Total SSB intake (g/day) was calculated and participants were categorized into light drinkers (SSB intake < 200 g/day) and moderate drinkers (SSB intake ≥ 200 g day).

### Biochemical parameters

Blood samples were collected after a minimum of 8 h of fasting and used on the day of sample collection to measure serum uric acid and creatinine (Cr) concentrations. Serum uric acid concentration (mg dL^-1^) was measured by the enzymatic uricase method using commercially available reagents (SICDIA L UA, Shin Yang Pharm., Seoul, Korea) [28]. Serum Cr levels were measured using the kinetic alkaline picrate (Jaffe’s) method with the Roche creatinine Jaffe reagent (Roche Diagnostics Limited, East Sussex, UK) [29]. We calculated eGFR using the revised Schwartz estimate: eGFR (mL min^-1^ 1.73 m^-2^) = 0.413 × height (cm)/serum Cr (mg dL^-1^) [30].

### Measurements of urinary BPA, BPS, and BPF levels

Spot urine samples were collected in the morning, sent to the laboratory (SMARTIVE Co., Institute for Life and Environmental Technology, Seoul, Korea) where the chemical analysis for urinary environmental phenols of the Korean national biomonitoring program (Korean National Environmental Health Survey, KoNEHS) is conducted. All urine samples were collected in alcohol-washed specimen cups and stored at −70°C until analysis. The analytical procedure has followed the previously reported methods [31, 32]. Urine specimens mixed with standard solutions were incubated overnight with β-glucuronidase/sulfatase (Sigma-Aldrich, Merck, NJ, US) and sodium acetate at 37°C. Next, the solid-phase extraction (Strata-X 33u Polymeric Reversed Phase 96-well plate) was performed using cartridges with acetonitrile (2 mL) and distilled water (2 mL), and the resulting extract (1 mL) of BPA, BPS, and BPF was eluted using methanol. Urinary levels of BPA, BPS, and BPF were quantified using the ultra-high-performance liquid chromatography–tandem mass spectrometry method (Agilent 6490 Triple Quad LCMS; Agilent, Santa Clara, CA, USA). Analytical procedures followed a strict internal quality assurance protocol that involved measuring procedural blanks and performing internal quality control (QC) on urine samples in each batch of measurements. The internal QC included tests for linearity, accuracy, precision, and detection limit. In the linearity test, R^2^ was 0.999 in the calibration curve, which applied seven points of the concentration range in pooled urine. The accuracy test was performed using standard reference materials (National Institute of Standards & Technology, NIST 3672 –organic contaminants in smokers’ urine and NIST 3673 –organic contaminants in non-smokers’ urine) and yielded recovery rates of BPA of 103.3%. For the precision test, the intra- and inter-day coefficient of variation of five samples was calculated, and was ≤ 5% for all analytes of the three bisphenols. The limits of detection (LOD) was 0.212 μg L^-1^ for BPA, 0.020 μg L^-1^ for BPS, and 0.074 μg L^-1^ for BPF, respectively. S1 Table summarizes the method parameters for internal quality control. For the external QC, the laboratory partook in the German External Quality Assessment Scheme and has passed the 57th to 63rd assessments (2016–2019; urinary BPA).

### Statistical analysis

We calculated descriptive statistics for bisphenol levels, including range, percentiles, and geometric mean (GM). Urinary BPA level was natural log transformed (continuous variables) or categorized into quartiles (categorical variables) for subsequent analysis. Values below the LOD were assigned a value of LOD divided by the square root. Since BPS and BPF were detected in 41.9% and 23.5% of the urine samples, respectively, we grouped BPS exposure into three categories (non-detection: < LOD, 0.02 μg L^-1^ (n = 284) vs. medium BPS: 0.02–0.05 μg L^-1^ (n = 102) vs. high BPS: ≥ 0.05 μg L^-1^ (n = 103)) and BPF exposure into two categories (non-detection: < LOD, 0.07 μg L^-1^ vs. detection: ≥ LOD) for regression analysis.

The participants’ characteristics were analyzed using Student’s t-test for continuous variables, and the Chi-squared test was used for categorical variables. Linear regression analysis was performed for the uric acid concentrations. Although the interaction of sex with the effect of bisphenols on uric acid concentration or hyperuricemia was not significant (all *P* > 0.1), a sex-stratified analysis was also performed. A linear trend of estimates for bisphenol categories was also observed in regression models.

Base models were adjusted for age, sex, and urinary Cr level to account for urine dilution [33]. Potential covariates for inclusion were selected based on a review of previous literature [8, 34–36], including BMI z-scores, eGFR, dietary animal protein intake (g/day), SSB intake (light drinkers vs. moderate drinkers), physical activity (minutes/week), monthly household income (≤ or > 4,000,000 KRW), and environmental tobacco smoke exposure. The model 2 was further adjusted for variables that significantly improved the model fit (model comparison using the *anova* function in R), or those significantly associated with serum uric acid concentrations (*P* < 0.05, S2 Table) that included BMI z-scores and eGFR. We also developed a directed acyclic graph (DAG) to identify potential confounding variables (S1 Fig) which included age, sex, urinary Cr, SSB intake, and income (model 3). Finally, we examined the association between urinary bisphenol levels and serum uric acid concentrations, including all the covariates used in the models (model 4). All statistical analyses were performed using R Statistical Software package (version 3.6.0; R Foundation for Statistical Computing, Vienna, Austria), and values of *P* < 0.05 were considered statistically significant.

## Results

### Participant characteristics

Table 1 shows the baseline characteristics of the 489 children (251 boys and 238 girls). The mean age was 5.9 years (0.1 standard deviations [SD]). The mean BMI z-score was -0.14 (1.04) without sex differences. Overweight and obesity was observed in 38 (7.8%) and 21 (4.3%) children, respectively. Dietary energy (1534 vs. 1417 kcal/day, *P* < 0.001) and animal protein intake (35.1 vs. 28.6 g/day, *P* = 0.001) were higher in boys than in girls. Total SSB intake was 135.0 g/day, and 104 (21.3%) participants were moderate SSB drinkers with no differences between sexes. The mean physical activity was 223 minutes per week, and boys were more likely to be physically active than girls (256 vs. 187 min/week, *P* = 0.001). Most families (70.8%) had a monthly household income higher than 4,000,000 KRW (≒ 3333 US$). Mean serum uric acid concentration was 4.2 mg dL^-1^ (0.8 SD) with no differences between sexes. However, participants with detectable urinary BPS levels showed significantly higher serum uric acid concentrations (4.3 vs. 4.1 mg dL^-1^, *P* = 0.011) than participants in the non-detection group (S3 Table).

**Table 1:** Demographic characteristics of the 489 participants.

Variable	Total (N = 489)	Boys (n = 251)	Girls (n = 238)
Age, years	5.9 (0.1)	5.9 (0.1)	5.9 (0.1)
Height, cm	115.6 (4.4)	116.0 (4.6)^a^	115.0 (4.1)^a^
Weight, kg	21.1 (3.2)	21.3 (3.1)	20.8 (3.2)
Body mass index, kg m^-2^	15.7 (1.8)	15.7 (1.7)	15.7 (1.9)
Height z-score	0.31 (0.95)	0.30 (0.98)	0.31 (0.93)
Weight z-score	0.06 (0.99)	-0.02 (0.98)	0.14 (1.00)
Body mass index z-score	-0.14 (1.04)	-0.22 (1.01)	-0.06 (1.06)
Overweight, no. (%)	38 (7.8)	16 (6.4)	22 (9.2)
Obesity, no. (%)	21 (4.3)	8 (3.2)	13 (5.5)
Total energy intake, kcal/day	1476.7 (356.1)	1533.9 (372.2)^a^	1416.6 (328.6)^a^
Dietary animal protein intake, g/day	30.4 (11.5)	35.1 (12.4)^a^	28.6 (10.1)^a^
Total sugar-sweetened beverage intake, g/day	135.0 (101.4)	140.9 (110.9)	128.8 (90.1)
Moderate sugar-sweetened beverage drinkers (≥ 200 g/day), no. (%)	104 (21.3)	60 (23.9)	44 (18.5)
Physical activity time, min/week	222.6 (236.5)	256.2 (266.9)^a^	187.1 (193.8)^a^
Monthly household income (> 4,000K KRW), no. (%)	346 (70.8)	169 (67.3)	177 (74.4)
Environmental tobacco smoke exposure, no. (%)	114 (23.3)	64 (25.6)	50 (21.0)
Serum creatinine, mg dL^-1^	0.41 (0.05)	0.41 (0.05)	0.41 (0.05)
Urininary creatinine, mg dL^-1^	81.3 (42.0)	85.2 (41.9)^a^	77.1 (41.9)^a^
Estimated glomerular filtration rate, mL min^-1^ 1.73m^-2^	117.4 (19.6)	117.6 (17.0)	117.1 (22.1)
Serum uric acid, mg dL^-1^	4.2 (0.8)	4.2 (0.8)	4.1 (0.7)

Data were expressed as number (percentage) or mean (standard deviation).

^a^*P* < 0.05

### Urinary BPA, BPS, and BPF levels of 6-year-old children

Table 2 shows the distributions of urinary BPA, BPS, and BPF levels. The detection frequency of urinary BPA, BPS, and BPF was 99.8%, 41.9%, and 23.5%, respectively. The median level was 1.58 μg L^-1^ for BPA, while those of BPS and BPF were below the LOD (< 0.020 μg L^-1^ for BPS, < 0.074 μg L^-1^ for BPF). The GM was 1.63 μg L^-1^for BPA, 0.08 μg L^-1^ for BPS, and 0.16 for BPF, without significant sex differences. The distribution of urinary Cr-adjusted bisphenol levels is described in S4 Table, showing no sex differences.

**Table 2:** Summary of BPA, BPS, and BPF (μg L^-1^) in urine from 6-year-old children.

Bisphenol		LOD	Detection frequency, no. (%)	Range, min-max	25^th^ percentile	50^th^ percentile	75^th^ percentile	Geometric mean (SD)	*P*
BPA	Total	0.212	488 (99.8)	0.150–153.126	0.992	1.582	2.503	1.629 (2.452)	0.078
	Boys		250 (99.6)	0.150–153.126	1.001	1.623	2.737	1.746 (2.679)	
	Girls		238 (100.0)	0.150–14.648	0.986	1.476	2.394	1.515 (2.200)	
BPS	Total	0.020	205 (41.9)	<LOD-21.456	< LOD	< LOD	0.041	0.075 (3.770)^a^	0.664
	Boys		106 (42.2)	<LOD-21.456	< LOD	< LOD	0.041	0.078 (3.638)^a^	
	Girls		99 (41.6)	<LOD-17.730	< LOD	< LOD	0.040	0.072 (3.933)^a^	
BPF	Total	0.074	115 (23.5)	<LOD-2.310	< LOD	< LOD	< LOD	0.157 (2.567)^a^	0.250
	Boys		61 (24.3)	<LOD-2.310	< LOD	< LOD	< LOD	0.173 (2.566)^a^	
	Girls		54 (22.7)	<LOD-1.946	< LOD	< LOD	< LOD	0.141 (2.560)^a^	

BPA, bisphenol A; BPS, bisphenol S; BPF, bisphenol F; LOD, limit of detection

^a^Geometric mean and SD values of for BPS and BPF were calculated among samples with bisphenol levels ≥ LOD.

### Relationship between urinary BPA, BPS, and BPF levels and serum uric acid concentrations

Of the three bisphenols, urinary BPS level was positively associated with serum uric acid concentrations (Table 3). In base model, which controlled for age, sex, and urinary creatinine levels, serum uric acid concentration was positively associated with urinary BPS levels (*P*-value for trend = 0.007). When the multivariate-adjusted model (model 2) was constructed including age, sex, urinary Cr, BMI z-scores, and estimated glomerular filtration rate (eGFR), the high BPS group showed significantly higher serum uric acid concentrations (by 0.26 mg dL^-1^, *P* = 0.003) than the non-detection group. It was noted that, as levels of BPS exposure rose, serum uric acid concentrations increased after adjustment for covariates (*P*-value for trend = 0.002). When stratified by sex, the association was significant in boys (*P*-value for trend < 0.001) but not girls. For boys, the high BPS group showed higher serum uric concentrations (by 0.43 mg dL^-1^, *P* < 0.001) than the non-detection group after adjusting for covariates. The association between urinary BPS and serum uric acid concentration remained robust after adjustment for covariates selected from DAG (model 3, S5 Table) or all other known covariates including age, sex, urinary Cr, BMI z-scores, eGFR, dietary animal protein intake, SSB intake, weekly minutes of physical activity, monthly household income, and environmental tobacco smoke exposure (model 4, S6 Table). However, for BPA and BPF levels, no significant associations with serum uric acid concentrations were observed.

**Table 3:** Association of urinary BPA, BPS, and BPF levels (μg L^-1^) with serum uric acid concentrations (mg dL^-1^).

Variables (concentration range)		N	Total (ß, 95% CI)		Boys (ß, 95% CI)		Girls (ß, 95% CI)	
			Base model	Model 2	Base model	Model 2	Base model	Model 2
Log-transformed BPA		489	0.03 (-0.05, 0.12)	0.04 (-0.04, 0.12)	0.01 (-0.10, 0.12)	0.01 (-0.09, 0.12)	0.06 (-0.07, 0.19)	0.08 (-0.05, 0.22)
BPA category	Q1 (< 0.99)	122	0 [Reference]	0 [Reference]	0 [Reference]	0 [Reference]	0 [Reference]	0 [Reference]
	Q2 (0.99–1.58)	122	0.03 (-0.16, 0.22)	2.25 (-1.09, 5.60)	0.03 (-0.26, 0.32)	-0.02 (-0.31, 0.26)	0.04 (-0.21, 0.30)	0.07 (-0.19, 0.32)
	Q3 (1.58–2.50)	122	0.12 (-0.08, 0.31)	0.03 (-0.16, 0.22)	0.03 (-0.25, 0.31)	0.05 (-0.22, 0.33)	0.23 (-0.04, 0.50)	0.23 (-0.04, 0.50)
	Q4 (≥ 2.50)	123	0.04 (-0.17, 0.25)	0.13 (-0.06, 0.33)	0.06 (-0.24, 0.35)	0.07 (-0.22, 0.36)	0.01 (-0.28, 0.30)	0.06 (-0.23, 0.35)
	*P* trend		0.527	0.351	0.723	0.552	0.594	0.425
BPS category	ND (< 0.02)	284	0 [Reference]	0 [Reference]	0 [Reference]	0 [Reference]	0 [Reference]	0 [Reference]
	Medium BPS (0.02–0.05)	102	0.08 (-0.09, 0.25)	0.11 (-0.06, 0.28)	0.16 (-0.09, 0.41)	0.19 (-0.05, 0.43)	-0.01 (-0.25, 0.22)	0.02 (-0.21, 0.25)
	High BPS (≥ 0.05)	103	0.25 (0.07, 0.42)^a^	0.26 (0.09, 0.43)^a^	0.41 (0.16, 0.66)^a^	0.43 (0.19, 0.67)^b^	0.06 (-0.19, 0.30)	0.07 (-0.17, 0.31)
	*P* trend		0.007	0.002	0.001	< 0.001	0.696	0.555
BPF category	ND (< 0.07)	374	0 [Reference]	0 [Reference]	0 [Reference]	0 [Reference]	0 [Reference]	0 [Reference]
	Detection (≥ 0.07)	115	0.00 (-0.16, 0.16)	0.02 (-0.15, 0.18)	-0.05 (-0.28, 0.18)	-0.01 (-0.24, 0.22)	0.06 (-0.17, 0.29)	0.06 (-0.17, 0.29)

BPA, bisphenol A; BPS, bisphenol S; BPF, bisphenol F; Q1, quartile 1; Q2, quartile 2; Q3, quartile 3; Q4, quartile 4; ND; non-detection

Base model was adjusted for age, sex, and urinary creatinine levels.

Model 2 was adjusted for age, sex, urinary creatinine levels, body mass index z-scores, and estimated glomerular filtration rate.

^a^*P* < 0.01;

^b^*P* < 0.001

## Discussion

Between 2015 and 2017, the BPA, BPS, and BPF urine levels of the 6-year-old Korean children in this study were 99.8%, 41.9%, and 23.5%, respectively. Of the three bisphenols, urinary BPS levels were significantly associated with serum uric acid concentrations. The greater the BPS exposure, the higher the serum uric acid concentrations; however, the increasing effect of BPS on uric acid concentration was significant in boys, but not girls.

Here, we found comparatively higher BPA concentrations (median, 1.58 μg L^-1^; GM, 1.63 μg L^-1^) than BPS (median, < 0.02 μg L^-1^; GM, 0.08 μg L^-1^) or BPF (median, < 0.07 μg L^-1^; GM, 0.16 μg L^-1^) concentrations. Urinary BPS levels in this cohort were comparable to previous reports on Korean adults (42.0% detection rate with median, 0.01 μg L^-1^) [37], but much lower than those in other countries [37–40]. In a study in the United States, children aged 6–11 years between 2013–2014 showed similar BPA levels (median 1.34 μg L^-1^) but higher BPS (median, 0.27 μg L^-1^) and BPF levels (median, 0.27 μg L^-1^) [38] than did this study. As for Chinese children aged 3–11 years in 2015, the median urinary levels were 0.35 μg L^-1^ for BPA, 0.03 μg L^-1^ for BPS, and 0.19 μg L^-1^ for BPF [39], suggesting higher BPS and BPF exposure than in Korean children. The timing of BPA regulations and the release of BPA-free products are major determinants of BPS and BPF exposure levels between countries [14–18, 41]. In the United States and Japan, the use of BPA-free products has steadily increased since their introduction in the early 2000s [38, 41]. In Korea, BPA regulations were first applied to infant feeding bottles in 2011 [17], and then extended to all infant instruments, containers, and packaging materials in 2019 [42]. BPA was substituted with BPS for epoxy resin, polycarbonate plastics, and thermal papers, and water pipe–coating agents were replaced with BPF [41]. Although the urinary levels of BPS and BPF were low in Korean children during 2015–2017, the increasing use of BPA-free products warrants further investigation regarding their safety and health effects.

Risk factors for hyperuricemia have been of interest since hyperuricemia was linked to hypertension, metabolic syndrome, diabetes, kidney disease, and cardiovascular diseases in studies on adults [43, 44]. In pediatric populations, serum uric acid concentrations have been associated with abdominal obesity [4, 7], high blood pressure [6, 45–47], insulin resistance [4], and metabolic syndrome [4, 7, 46]. In our children’s cohort, BMI z-scores and eGFR, reflective of reflecting kidney function, were significantly related to serum uric acid concentrations (S2 Table). This is consistent with previous studies [8, 34–36] reporting age, sex, obesity, socioeconomic status, and lifestyle factors such as physical inactivity and animal protein or fructose consumption as risk factors. To the best of our knowledge, the relationship of BPA and its substitutes to serum uric acid concentrations has not been investigated in pediatric populations. Further studies should be conducted after adjusting for known risk factors of hyperuricemia.

Of the three bisphenols, BPS exposure was significantly associated with increase in serum uric acid levels among school-aged children. Although the mechanism remains unclear, bisphenols can affect uric acid metabolism [48–50]. The balance between hepatic synthesis by xanthine oxidoreductase (XO) and renal or intestinal elimination by uric acid transporters determines serum uric acid concentrations [1]. A previous animal study showed that BPA may induce hyperuricemia by activating hepatic XO activity through direct binding [48]. Another putative mechanism is that BPA may downregulate uric acid transporters in the kidney and intestine, such as adenosine triphosphate binding cassette subfamily G member 2 (ABCG2) [49], thereby contributing to hyperuricemia [51]. Bisphenols-induced oxidative stress in liver and renal endothelial cells can mediate hyperuricemia [1, 10, 50]. Nonetheless, only one adult study has been conducted for the effect of BPA exposure on hyperuricemia, reporting that baseline serum BPA levels predicted the development of hyperuricemia after 6 years of follow-up [52]. However, in this study, we did not find a significant association between BPA levels and uric acid levels. Neither epidemiological nor experimental studies have investigated the relationship between BPA substitutes and uric acid metabolism. Thus, the mechanism underlying the association between BPS and serum uric acid levels remains unknown. The human body presumably needs the antioxidant properties of uric acid to counteract BPS-induced oxidative stress [21, 53], thereby leading to an increase in uric acid levels. Although the effect size was small, this positive relationship between BPS exposure and uric acid concentration should be examined further.

We found a more significant relationship between BPS exposure and serum uric acid concentrations in boys than in girls. Men generally have higher XO activity [54] and less renal uric acid elimination ability due to the relatively lower expression of renal uric acid transporters such as ABCG2 and urate anion transporter 1 [55], leading to higher uric acid concentrations in men than in women. This sex-based difference in uric acid metabolism may support the higher susceptibility to bisphenol-induced hyperuricemia in boys.

This study had several limitations. First, its cross-sectional design cannot prove a causal relationship between bisphenols and serum uric acid concentrations. Second, bisphenol levels were measured in a single spot urine sample. Due to the short half-life of bisphenol analogs (6.2–6.4 hours for BPA and 6.8 hours for BPS) [56, 57], the spot urine sample has limited ability to capture intra-individual variability over time; nonetheless, a single spot urine sample may adequately reflect the population’s average BPA exposure [58]. A recent pediatric study also supported the notion that a single spot urine sample provides a reliable characterization of absolute and relative exposure in young children [59]. To eliminate within-day variations, urine samples were collected in the morning. Third, the low detection frequency of BPS and BPF limited our ability to assess linear relationships between continuous measures of exposure and outcome. In addition, we could not measure urinary urate concentrations to evaluate the fractional renal clearance of serum urate. This limited our ability to determine whether BPS exposure affects renal clearance of uric acid. Further longitudinal investigations of the correlations between BPA substitute exposure and health outcomes are required. To our knowledge, however, this study is the first to report on relationships of BPA, BPS, and BPF and serum uric acid concentrations in preschool children.

In conclusion, higher exposure to BPS was associated with increased serum uric acid concentration in 6-year-old children, although only significantly in boys. Considering the increasing use of BPS and the concerning effect of increase in uric acid levels on pediatric health outcomes, further prospective studies are needed to determine the possible health effects of BPA substitutes, and to elucidate the underlying mechanism thereof.

## Supporting information

S1 Appendix Environmental exposure, parental, and physical activity questionnaires (6-year-old). (DOCX)

S2 Appendix Food frequency questionnaire. (DOCX)

S1 Table Summary of method parameters for internal quality control. (DOCX)

S2 Table Covariates associated with serum uric acid concentrations (mg dL^-1^). (DOCX)

S3 Table Participant characteristics stratified by BPS and BPF detection in urine samples. (DOCX)

S4 Table Summary of creatinine-adjusted BPA, BPS, and BPF (μg g^-1^ Cr) in urine from 6-year-old children. (DOCX)

S5 Table Association of urinary BPA, BPS, and BPF levels (μg L^-1^) with serum uric acid concentrations (mg dL^-1^) adjusted for covariates selected by directed acyclic graph (DAG) (model 3). (DOCX)

S6 Table Association of urinary BPA, BPS, and BPF levels (μg L^-1^) with serum uric acid concentrations (mg dL^-1^) after adjusting for all possible covariates (model 4). (DOCX)

S1 Fig Directed acyclic graph showing associations between bisphenol exposures, covariates, and serum uric acid concentrations. Directed acyclic graphs (DAGs) show the hypothesized causal relationship between bisphenol exposure (yellow circle), covariates, and serum uric acid concentrations (‘I’ in a blue circle). The proposed adjustment variables are indicated by white circles and the proposed adjustment variables in the model are indicated by blue circles.
(TIF)
