# Supplementary material for: Relationship between bisphenol A, bisphenol S, and bisphenol F and serum uric acid concentrations among school-aged children
Source: PLoS One. 2022 Jun 16;17(6):e0268503. doi: 10.1371/journal.pone.0268503 (PMC9202957; doi:10.1371/journal.pone.0268503)
Supplement: S1 Appendix — (DOCX) [file pone.0268503.s001.docx]

**English version**

**Environmental exposure questionnaire (6-year-old)**

Does anyone smoke at home?

| - Yes 🡪 If yes, how many people? ___________ |
| --- |
| - No |

**Parental questionnaire (6-year-old)**

What is your household income?

① Less than 2,000,000 KRW

② 2,000,000 – 4,000,000 KRW

③ 4,000,000 – 6,000,000 KRW

④ 6,000,000 or more KWR

**Physical activity questionnaire (6-year-old)**

1. In the past week, how many days did you engage in vigorous physical activity for more than 10 min?

□ None (0 days) □ 1 day □ 2 days □ 3 days □ 4 days □ 5 days □ 6 days □ 7 days

| Examples of vigorous physical activity: jogging (running), soccer, baseball, jumping rope, judo, taekwondo, kendo, rock climbing, hiking, aerobic dance, tennis, squash, hockey, roller, skating, biking (fast), swimming (fast), carrying heavy objects |
| --- |

How many minutes a day do you usually engage in these vigorous activities?

( ) h ( ) min/day

2. In the past week, how many days did you engage in moderate physical activity for more than 10 min?

□ None (0 days) □ 1 d □ 2 days □ 3 days □ 4 days □ 5 days □ 6 days □ 7 days

| Examples of moderate physical activity: volleyball, badminton, ping-pong table tennis, swimming (slowly), tennis doubles, folk dancing, carrying light objects (excluding walking) |
| --- |

How many minutes a day do you usually engage in these moderate-intensity activities?

( ) h ( ) min/day

**Korean version**

**환경요인노출 설문조사 (6세용)**

집 안에서 담배를 피우는 사람이 있습니까?

| - 예 🡪 있다면 몇 명입니까? ___________명 |
| --- |
| - 아니오 |

**부모의 건강상태 조사 (6세용)**

귀하 가정의 월 평균수입(가구 전체 소득)은 어느 정도 되십니까?

1. 200만원 미만
2. 200만원 이상 ~ 400만원 미만
3. 400만원 이상 ~ 600만원 미만
4. 600만원 이상

**신체활동 설문지 (6세용)**

1. 지난 1주일 동안 평소보다 몸이 매우 힘들거나 숨이 많이 가쁜 격렬한 신체활동을 10분 이상 한 날은 며칠입니까?

□ 안 함 (0일) □ 1일 □ 2일 □ 3일 □ 4일 □ 5일 □ 6일 □ 7일 (매일)

| 격렬한 신체활동의 예) 조깅(달리기), 축구, 농구, 줄넘기, 유도, 태권도, 검도, 암벽등반, 등산, 에어로빅 댄스, 단식 테니스, 스쿼시, 하키, 롤러 스케이트, 자전거(빠르게), 수영(빠르게), 무거운 물건 나르기 등 |
| --- |

이러한 격렬한 신체활동을 한 날, 보통 하루에 몇 분간 했습니까?

하루에 ( ) 시간 ( ) 분

2. 지난 1주일 동안 평소보다 몸이 조금 힘들거나 숨이 약간 가쁜 중등도 신체활동을 10분 이상 한 날은 며칠입니까?

□ 안 함 (0일) □ 1일 □ 2일 □ 3일 □ 4일 □ 5일 □ 6일 □ 7일 (매일)

| 중등도 신체활동의 예) 배구, 배드민턴, 탁구, 수영(천천히), 복식 테니스, 볼륨/포크 댄스, 가벼운 물건 나르기 등 (걷기 제외) |
| --- |

이러한 중등도 신체활동을 한 날, 보통 하루에 몇 분간 했습니까?

하루에 ( ) 시간 ( ) 분
