# Supplementary material for: Relationship between bisphenol A, bisphenol S, and bisphenol F and serum uric acid concentrations among school-aged children
Source: PLoS One. 2022 Jun 16;17(6):e0268503. doi: 10.1371/journal.pone.0268503 (PMC9202957; doi:10.1371/journal.pone.0268503)
Supplement: S2 Appendix — (DOCX) [file pone.0268503.s002.docx]

**Food frequency questionnaire**

※ Please mark how often your child has consumed the following categories of foods or drinks during the past year. Please mark every food item accurately without leaving out any foods.

□ For the frequency of usual food intake, please indicate (✓) how often your child consumed a food, on average, during the past year. If your child consumed a food less than once per month, please choose “Never.”

□ Typical portion size

Compare your child’s portion with the standard portion size.

If it was less than 50% of the standard portion size, please mark “Less.”

If it was 50–150% of the standard portion size, please mark “Standard.”

If it was more than 150% of the standard portion size, please mark “More.”

If you do not know the standard portion size, compare the amount your child ate with friends of same age group.

□ For foods containing more than one item, mark the amount consumed of at least one of them.

□ For seasonal foods, consider how often your child ate them in that season, even if your child does not eat them currently.

**Sample data collection table**

| **Food group** | **Average consumption last year** | | | | | | | | | **Average portion consumed at a time** | | | |
| --- | --- | --- | --- | --- | --- | --- | --- | --- | --- | --- | --- | --- | --- |
|  | **Never** | **Per month** | | **Per week** | | | **Per day** | | |  |  |  |  |
|  |  | **1** | **2-3** | **1-2** | **3-4** | **5-6** | **1** | **2** | **3+** | **Standard portion size** | **Less** | **Standard** | **More** |
| **Staple food** | | | | | | | | | | | | | |
| Rice |  |  |  |  |  |  |  |  |  | 1 child’s bowl |  |  |  |
| Bread |  |  |  |  |  |  |  |  |  | 1 piece (50 g) |  |  |  |
| **Beef** | | | | | | | | | | | | | |
| **Pork** | | | | | | | | | | | | | |
| **Chicken/eggs** | | | | | | | | | | | | | |
| **Fish** | | | | | | | | | | | | | |
| **Pulses** | | | | | | | | | | | | | |
| **Kimchi group** | | | | | | | | | | | | | |
| **Vegetables** | | | | | | | | | | | | | |
| **Seaweeds** | | | | | | | | | | | | | |
| **Dairy products** | | | | | | | | | | | | | |
| **Fat and sweets** | | | | | | | | | | | | | |
| **Fruits** | | | | | | | | | | | | | |
| **Fruit juice** | | | | | | | | | | | | | |
| **Drinks** | | | | | | | | | | | | | |

**Sample food frequency questionnaire for sugar-sweetened beverages**

| **Food group** | **Average use last year** | | | | | | | | | **Average portion consumed at a time** | | | |
| --- | --- | --- | --- | --- | --- | --- | --- | --- | --- | --- | --- | --- | --- |
|  | **Never** | **Per month** | | **Per week** | | | **Per day** | | |  |  |  |  |
|  |  | **1** | **2-3** | **1-2** | **3-4** | **5-6** | **1** | **2** | **3+** | **Standard portion size** | **Less** | **Standard** | **More** |
| **Dairy products** | | | | | | | | | | | | | |
| Flavored milk (chocolate, banana, strawberry, etc) |  |  |  |  |  |  |  |  |  | 1 cup (200 mL) |  |  |  |
| Soymilk |  |  |  |  |  |  |  |  |  | 1 cup (200 mL) |  |  |  |
| Fermented milk |  |  |  |  |  |  |  |  |  | 1 serving (150 g) |  |  |  |
| Yogurt drink |  |  |  |  |  |  |  |  |  | 1 serving |  |  |  |
| **Fruit juice** | | | | | | | | | | | | | |
| Orange juice |  |  |  |  |  |  |  |  |  | 1 cup (200 mL) |  |  |  |
| Tomato juice |  |  |  |  |  |  |  |  |  | 1 cup (200 mL) |  |  |  |
| Other juice (apple, grape, etc.) |  |  |  |  |  |  |  |  |  | 1 cup (200 mL) |  |  |  |
| **Drinks** | | | | | | | | | | | | | |
| Cinnamon punch, sweet rice drink |  |  |  |  |  |  |  |  |  | 1 cup (200 mL) |  |  |  |
| Drinking chocolate |  |  |  |  |  |  |  |  |  | 1 cup (200 mL) |  |  |  |
| Carbonated beverages (Coke, Sprite, etc.) |  |  |  |  |  |  |  |  |  | 1 cup (200 mL) |  |  |  |
| Sports and energy drinks |  |  |  |  |  |  |  |  |  | 1 cup (200 mL) |  |  |  |

**Korean version**

**식품섭취 빈도조사**

※ 귀하의 자녀가 지난 1년 동안 다음 식품을 얼마나 자주 섭취했는지를 해당하는 곳에 표시하십시오. 한 가지 식품도 빼놓지 말고 정확하게 모두 표시해야 합니다.

□ 평상시 음식(식품) 섭취 빈도는 지난 1년 동안 평균적으로 얼마나 자주 먹었는지를 해당하는 곳에 ✓ 표시해 주세요. 평균적으로 한달에 한 번도 먹지 않는 경우는 ‘거의 안먹음’에 표시해 주세요.

□ 평상 시 먹는 양은

- 귀하의 자녀가 섭취한 평균 1회 분량을 기준분량과 비교하여

1/2 이하이면 ‘더 적음’ 란에

기준분량의 1/2 이상에서 1.5배 이하이면 ‘기준분량’란에

1.5배 이상이면 ‘더 많음’ 란에 ✓ 표시합니다.

기준분량을 잘 알지 못하면 먹은 양을 같은 연령대의 친구들과 비교하여 ‘더 적음’, ‘기준분량’, ‘더 많음’ 중에서 선택하세요.

□ 한 가지 항목에 여러 가지 식품이 함께 제시된 것은 그 중 하나라도 먹는 것이 있다면 그 식품에 대해 작성하십시오.

□ 1년 중 특정 계절에만 먹는 식품은 현재는 먹지 않더라도 그 계절에 얼마나 자주 먹는지 생각하여 작성하십시오.

**식품 섭취 빈도 조사 표 예시**

| **식품군** | **지난 1년간 평균 섭취빈도** | | | | | | | | | **평균 1회 섭취분량** | | | |
| --- | --- | --- | --- | --- | --- | --- | --- | --- | --- | --- | --- | --- | --- |
|  | **거의**  **안먹음** | **한 달** | | **일 주 일** | | | **하 루** | | |  |  |  |  |
|  |  | **1회** | **2-3회** | **1-2회** | **3-4회** | **5-6회** | **1회** | **2회** | **3회**  **이상** | **기준분량** | **더**  **적음** | **기준**  **분량** | **더**  **많음** |
| **주식류** | | | | | | | | | | | | | |
| 쌀밥 | □0 | □1 | □2 | □3 | □4 | □5 | □6 | □7 | □8 | 어린이용 1공기 |  |  |  |
| 식빵, 토스트 | □0 | □1 | □2 | □3 | □4 | □5 | □6 | □7 | □8 | 큰 것 1쪽(50g) |  |  |  |
| **쇠고기** | | | | | | | | | | | | | |
| **돼지고기** | | | | | | | | | | | | | |
| **닭고기/난류** | | | | | | | | | | | | | |
| **생선류** | | | | | | | | | | | | | |
| **콩류** | | | | | | | | | | | | | |
| **김치류** | | | | | | | | | | | | | |
| **채소류** | | | | | | | | | | | | | |
| **해조류** | | | | | | | | | | | | | |
| **우유 및 유제품** | | | | | | | | | | | | | |
| **유지 및 당류** | | | | | | | | | | | | | |
| **과일류** | | | | | | | | | | | | | |
| **과일 주스** | | | | | | | | | | | | | |
| **음료류** | | | | | | | | | | | | | |

**가당 음료 식품 섭취 빈도 조사**

| **식품군** | **지난 1년간 평균 섭취빈도** | | | | | | | | | **평균 1회 섭취분량** | | | |
| --- | --- | --- | --- | --- | --- | --- | --- | --- | --- | --- | --- | --- | --- |
|  | **거의**  **안먹음** | **한 달** | | **일 주 일** | | | **하 루** | | |  |  |  |  |
|  |  | **1회** | **2-3회** | **1-2회** | **3-4회** | **5-6회** | **1회** | **2회** | **3회**  **이상** | **기준분량** | **더**  **적음** | **기준**  **분량** | **더**  **많음** |
| **우유 및 유제품** | | | | | | | | | | | | | |
| 향우유  (초코렛/바나나/ 딸기우유 등) | □0 | □1 | □2 | □3 | □4 | □5 | □6 | □7 | □8 | 1컵(200ml) |  |  |  |
| 두유 | □0 | □1 | □2 | □3 | □4 | □5 | □6 | □7 | □8 | 1컵(200ml) |  |  |  |
| 발효유  (불가리스, 요플레 등) | □0 | □1 | □2 | □3 | □4 | □5 | □6 | □7 | □8 | 1개(150g) |  |  |  |
| 유산균 음료  (한국야쿠르트,  이오 등) | □0 | □1 | □2 | □3 | □4 | □5 | □6 | □7 | □8 | 1개 |  |  |  |
| **과일 주스** | | | | | | | | | | | | | |
| 오렌지주스 | □0 | □1 | □2 | □3 | □4 | □5 | □6 | □7 | □8 | 1컵(200ml) |  |  |  |
| 토마토주스 | □0 | □1 | □2 | □3 | □4 | □5 | □6 | □7 | □8 | 1컵(200ml) |  |  |  |
| 기타주스  (사과, 포도 등) | □0 | □1 | □2 | □3 | □4 | □5 | □6 | □7 | □8 | 1컵(200ml) |  |  |  |
| **음료류** | | | | | | | | | | | | | |
| 수정과, 식혜 | □0 | □1 | □2 | □3 | □4 | □5 | □6 | □7 | □8 | 1컵(200ml) |  |  |  |
| 코코아 | □0 | □1 | □2 | □3 | □4 | □5 | □6 | □7 | □8 | 1잔(100ml) |  |  |  |
| 일반 탄산음료  (콜라, 사이다 등) | □0 | □1 | □2 | □3 | □4 | □5 | □6 | □7 | □8 | 1컵(200ml) |  |  |  |
| 기능성 ‧ 이온음료 (포카리스웨트, 파워에이드, 미에로 화이바 등) | □0 | □1 | □2 | □3 | □4 | □5 | □6 | □7 | □8 | 1컵(200ml) |  |  |  |
