# Supplementary material for: Relationship between bisphenol A, bisphenol S, and bisphenol F and serum uric acid concentrations among school-aged children
Source: PLoS One. 2022 Jun 16;17(6):e0268503. doi: 10.1371/journal.pone.0268503 (PMC9202957; doi:10.1371/journal.pone.0268503)
Supplement: S1 Table — (DOCX) [file pone.0268503.s003.docx]

**S1 Table. Summary of method parameters for internal quality control**

| **Compound** | **Linearity** | | **Precision** | **Accuracy** | **Detection limit** | |
| --- | --- | --- | --- | --- | --- | --- |
|  | **Dynamic range of calibration curve (μg L^-1^)** | **R^2^ for calibration curve** | **Mean CV (%)^a^** | **Mean recovery rate (%)^a^** | **LOD (μg L^-1^)** | **LOQ (μg L^-1^)** |
| **BPA** | 0.50-50.00 | 0.9987 | 4.8 | 103.3 | 0.212 | 0.636 |
| **BPS** | 0.10-50.00 | 0.9987 | 4.4 | 99.77 | 0.020 | 0.060 |
| **BPF** | 0.05-50.00 | 0.9993 | 4.7 | 95.97 | 0.074 | 0.222 |

CV, coefficient of variation, LOD, limit of detection; LOQ, limit of quantitation; BPA, bisphenol A; BPS, bisphenol S; BPF, bisphenol F

^a^ Recovery rate and coefficient of variation was calculated using 15 samples
