# Supplementary material for: Relationship between bisphenol A, bisphenol S, and bisphenol F and serum uric acid concentrations among school-aged children
Source: PLoS One. 2022 Jun 16;17(6):e0268503. doi: 10.1371/journal.pone.0268503 (PMC9202957; doi:10.1371/journal.pone.0268503)
Supplement: S2 Table — (DOCX) [file pone.0268503.s004.docx]

**S2 Table. Covariates associated with serum uric acid concentrations (mg dL^-1^)**

| Variables | N | ß (95% Confidence interval) | *P* value |
| --- | --- | --- | --- |
| Age, years | 489 | 0.48 (-0.08, 1.05) | 0.091 |
| Sex (male) | 489 | 0.08 (-0.06, 0.21) | 0.259 |
| Body mass index z-score | 489 | 0.10 (0.04, 0.16) | 0.003 |
| Dietary animal protein intake, g/day | 488 | 0.00 (0.00, 0.01) | 0.192 |
| Moderate sugar-sweetened beverage drinker (≥ 200 g/day) | 489 | 0.07 (-0.09, 0.24) | 0.392 |
| Physical activity time, min/week^a^ | 489 | -0.02 (-0.05, 0.02) | 0.323 |
| Monthly household income (> 4,000K KRW) | 489 | 0.02 (-0.13, 0.16) | 0.823 |
| Environmental tobacco smoke exposure (yes) | 489 | -0.12 (-0.27, 0.04) | 0.151 |
| Urinary creatinine, mg dL^-1^ | 489 | 0.00 (0.00, 0.00) | < 0.001 |
| Estimated glomerular filtration rate, mL min^-1^ 1.73m^-2^ | 489 | -0.01 (-0.01, 0.00) | < 0.001 |

For each covariate, the linear regression analysis was performed to evaluate the association with serum uric acid concentrations.

^a^Log-transformed for analysis
