# Supplementary material for: Relationship between bisphenol A, bisphenol S, and bisphenol F and serum uric acid concentrations among school-aged children
Source: PLoS One. 2022 Jun 16;17(6):e0268503. doi: 10.1371/journal.pone.0268503 (PMC9202957; doi:10.1371/journal.pone.0268503)
Supplement: S3 Table — (DOCX) [file pone.0268503.s005.docx]

**S3 Table. Participant characteristics stratified by BPS and BPF detection in urine samples**

| Variable | BPS | | BPF | |
| --- | --- | --- | --- | --- |
|  | Non-detection  (n = 284) | Detection  (n = 205) | Non-detection  (n = 374) | Detection  (n = 115) |
| Boys, no (%) | 145 (51.1) | 106 (51.7) | 190 (50.8) | 61 (53.0) |
| Age, years | 5.9 (0.1) | 5.9 (0.1) | 5.9 (0.1) | 5.9 (0.1) |
| Height, cm | 115.4 (4.3) | 115.7 (4.4) | 115.4 (4.4) | 116.0 (4.2) |
| Weight, kg | 20.9 (2.9) | 21.3 (3.5) | 20.9 (3.1) | 21.5 (3.6) |
| Body mass index, kg m^-2^ | 15.6 (1.5) | 15.8 (2.1) | 15.7 (1.6) | 16.0 (2.1) |
| Height z-score | 0.28 (0.95) | 0.34 (0.96) | 0.28 (0.96) | 0.40 (0.92) |
| Weight z-score | 0.02 (0.97) | 0.11 (1.03) | 0.02 (0.99) | 0.20 (0.99) |
| Body mass index z-score | -0.16 (0.96) | -0.12 (1.14) | -0.18 (1.03) | -0.03 (1.06) |
| Overweight, no. (%) | 21 (7.4) | 17 (8.3) | 28 (7.5) | 10 (8.7) |
| Obesity, no. (%) | 9 (3.2) | 12 (5.9) | 15 (4.0) | 6 (5.2) |
| Total energy intake, kcal/day | 1467.6 (365.3) | 1489.3 (343.4) | 1466.3 (358.5) | 1510.4 (347.6) |
| Dietary animal protein intake, g/day | 30.4 (11.9) | 30.4 (11.0) | 30.1 (11.7) | 31.2 (10.8) |
| Total sugar-sweetened beverage intake, g/day | 131.1 (98.2) | 140.5 (105.6) | 135.7 (102.8) | 132.9 (96.9) |
| Moderate sugar-sweetened beverage drinker (≥ 200g/day), no. (%) | 54 (19.0) | 50 (24.4) | 81 (21.7) | 23 (20.0) |
| Physical activity time, min/week | 214.8 (227.8) | 233.3 (248.2) | 227.1 (224.9) | 207.9 (207.1) |
| Monthly household income (> 4,000K KRW) no. (%) | 202 (71.1) | 144 (70.2) | 263 (70.3) | 83 (72.2) |
| Environmental tobacco smoke exposure, no. (%) | 71 (25.0) | 43 (21.0) | 92 (24.6) | 22 (19.1) |
| Serum creatinine, mg dL^-1^ | 0.42 (0.05) | 0.41 (0.06) | 0.42 (0.05) | 0.41 (0.06) |
| Urinary creatinine, mg dL^-1^ | 73.6 (38.7)^a^ | 91.9 (44.2)^a^ | 74.8 (41.1)^a^ | 102.2 (38.0)^a^ |
| Estimated glomerular filtration rate, mL min^-1^ 1.73m^-2^ | 116.0 (14.8) | 119.3 (24.7) | 116.7 (19.7) | 119.4 (19.4) |
| Serum uric acid, mg dL^-1^ | 4.1 (0.7)^a^ | 4.3 (0.8)^a^ | 4.1 (0.8) | 4.2 (0.8) |

Data were expressed as number (percentage) or mean (standard deviation).

BPS, bisphenol S; BPF, bisphenol F

^a^*P* < 0.05
