# Supplementary material for: Relationship between bisphenol A, bisphenol S, and bisphenol F and serum uric acid concentrations among school-aged children
Source: PLoS One. 2022 Jun 16;17(6):e0268503. doi: 10.1371/journal.pone.0268503 (PMC9202957; doi:10.1371/journal.pone.0268503)
Supplement: S4 Table — (DOCX) [file pone.0268503.s006.docx]

**S4 Table. Summary of creatinine-adjusted BPA, BPS, and BPF (μg g^-1^ Cr) in urine from 6-year-old children**

| Bisphenol |  | Range, min-max | 25^th^ percentile | 50^th^ percentile | 75^th^ percentile | Geometric mean (SD) | *P* |
| --- | --- | --- | --- | --- | --- | --- | --- |
| BPA | Total | 0.138-145.640 | 1.375 | 2.189 | 3.864 | 2.349 (2.320) | 0.106 |
|  | Boys | 0.138-145.640 | 1.307 | 2.102 | 3.793 | 2.365 (2.497) |  |
|  | Girls | 0.206-19.394 | 1.463 | 2.252 | 3.892 | 2.332 (2.136) |  |
| BPS | Total | < LOD-18.476 | < LOD | < LOD | 0.055 | 0.095 (3.907)^a^ | 0.764 |
|  | Boys | < LOD-18.476 | < LOD | < LOD | 0.049 | 0.091 (3.934)^a^ |  |
|  | Girls | < LOD-12.227 | < LOD | < LOD | 0.058 | 0.099 (3.900)^a^ |  |
| BPF | Total | < LOD-6.356. | < LOD | < LOD | < LOD | 0.167 (2.736)^a^ | 0.572 |
|  | Boys | < LOD-1.566 | < LOD | < LOD | < LOD | 0.180 (2.630)^a^ |  |
|  | Girls | < LOD-6.536 | < LOD | < LOD | < LOD | 0.154 (2.865)^a^ |  |

BPA, bisphenol A; BPS, bisphenol S; BPF, bisphenol F; LOD, limit of detection

^a^Geometric mean and SD values of for BPS and BPF were calculated among samples with bisphenol levels ≥ LOD.
