# Supplementary material for: Relationship between bisphenol A, bisphenol S, and bisphenol F and serum uric acid concentrations among school-aged children
Source: PLoS One. 2022 Jun 16;17(6):e0268503. doi: 10.1371/journal.pone.0268503 (PMC9202957; doi:10.1371/journal.pone.0268503)
Supplement: S5 Table — (DOCX) [file pone.0268503.s007.docx]

**S5 Table. Association of urinary BPA, BPS, and BPF levels (μg L^-1^) with serum uric acid concentrations (mg dL^-1^) adjusting for covariates selected by directed acyclic graph (DAG) (model 3)**

| Variables (concentration range) | | N | Total (ß, 95% CI) | Boys (ß, 95% CI) | Girls (ß, 95% CI) |
| --- | --- | --- | --- | --- | --- |
| Log-transformed BPA | | 489 | 0.03 (-0.05, 0.12) | 0.01 (-0.10, 0.12) | 0.06 (-0.08, 0.19) |
| BPA category | Q1 (< 0.99) | 122 | 0 [Reference] | 0 [Reference] | 0 [Reference] |
|  | Q2 (0.99-1.58) | 122 | 0.03 (-0.17, 0.22) | 0.02 (-0.27, 0.31) | 0.03 (-0.23, 0.29) |
|  | Q3 (1.58-2.50) | 122 | 0.12 (-0.08, 0.31) | 0.02 (-0.27, 0.30) | 0.23 (-0.04, 0.51) |
|  | Q4 (≥ 2.50) | 123 | 0.03 (-0.18, 0.24) | 0.05 (-0.25, 0.35) | -0.01 (-0.30, 0.29) |
|  | *P* trend | | 0.563 | 0.766 | 0.617 |
| BPS category | ND (< 0.02) | 284 | 0 [Reference] | 0 [Reference] | 0 [Reference] |
|  | Medium BPS (0.02-0.05) | 102 | 0.08 (-0.09, 0.25) | 0.16 (-0.09, 0.41) | -0.02 (-0.25, 0.22) |
|  | High BPS (≥ 0.05) | 103 | 0.25 (0.07, 0.42)^a^ | 0.41 (0.16, 0.66)^a^ | 0.05 (-0.19, 0.30) |
|  | *P* trend | | 0.007 | 0.001 | 0.737 |
| BPF category | ND (< 0.07) | 374 | 0 [Reference] | 0 [Reference] | 0 [Reference] |
|  | Detection (≥ 0.07) | 115 | 0.00 (-0.16, 0.16) | -0.05 (-0.29, 0.18) | 0.06 (-0.17, 0.29) |

BPA, bisphenol A; BPS, bisphenol S; BPF, bisphenol F; Q1, quartile 1; Q2, quartile 2; Q3, quartile 3; Q4, quartile 4; ND; non-detection

Models were adjusted for age, sex, urinary creatinine levels, sugar-sweetened beverage intake (light vs. moderate drinkers), and monthly household income.

^a^*P* < 0.01
