# Supplementary material for: Relationship between bisphenol A, bisphenol S, and bisphenol F and serum uric acid concentrations among school-aged children
Source: PLoS One. 2022 Jun 16;17(6):e0268503. doi: 10.1371/journal.pone.0268503 (PMC9202957; doi:10.1371/journal.pone.0268503)
Supplement: S6 Table — (DOCX) [file pone.0268503.s008.docx]

**S6 Table. Association of urinary BPA, BPS, and BPF levels (μg L^-1^) with serum uric acid concentrations (mg dL^-1^) after adjusting for all possible covariates (model 4)**

| Variables (concentration range) | | N | Total (ß, 95% CI) | Boys (ß, 95% CI) | Girls (ß, 95% CI) |
| --- | --- | --- | --- | --- | --- |
| Log-transformed BPA | | 488 | 0.04 (-0.04, 0.12) | 0.02 (-0.09, 0.12) | 0.08 (-0.05, 0.22) |
| BPA category | Q1 (< 0.99) | 122 | 0 [Reference] | 0 [Reference] | 0 [Reference] |
|  | Q2 (0.99-1.58) | 122 | 0.02 (-0.18, 0.21) | -0.07 (-0.36, 0.21) | 0.07 (-0.19 0.33) |
|  | Q3 (1.58-2.50) | 122 | 0.12 (-0.08, 0.31) | -0.02 (-0.30, 0.25) | 0.26 (-0.01, 0.53) |
|  | Q4 (≥ 2.50) | 122 | 0.06 (-0.15, 0.27) | 0.09 (-0.20, 0.38) | 0.05 (-0.25, 0.35) |
|  | *P* trend | | 0.391 | 0.529 | 0.413 |
| BPS category | ND (< 0.02) | 284 | 0 [Reference] | 0 [Reference] | 0 [Reference] |
|  | Medium BPS (0.02-0.05) | 101 | 0.11 (-0.06, 0.28) | 0.21 (-0.03, 0.45) | -0.01 (-0.24, 0.23) |
|  | High BPS (≥ 0.05) | 103 | 0.27 (0.10, 0.44)^a^ | 0.45 (0.22, 0.69)^b^ | 0.07 (-0.18, 0.31) |
|  | *P* trend | | 0.002 | < 0.001 | 0.627 |
| BPF category | ND (< 0.07) | 373 | 0 [Reference] | 0 [Reference] | 0 [Reference] |
|  | Detection (≥ 0.07) | 115 | 0.01 (-0.15, 0.18) | 0.02 (-0.21, 0.25) | 0.06 (-0.18, 0.29) |

BPA, bisphenol A; BPS, bisphenol S; BPF, bisphenol F; Q1, quartile 1; Q2, quartile 2; Q3, quartile 3; Q4, quartile 4; ND; non-detection

Models were adjusted for age, sex, urinary creatinine levels, body mass index z-scores, estimated glomerular filtration rate, dietary animal protein intake, sugar-sweetened beverage intake (light vs. moderate drinkers), weekly minutes for physical activities, monthly household income, and environmental tobacco smoke exposure. Data of dietary protein intake was missing in one participant.

^a^*P* < 0.01; ^b^*P* < 0.001
