# Supplementary figures and images for: Relationship between bisphenol A, bisphenol S, and bisphenol F and serum uric acid concentrations among school-aged children
Source: PLoS One. 2022 Jun 16;17(6):e0268503. doi: 10.1371/journal.pone.0268503 (PMC9202957; doi:10.1371/journal.pone.0268503)

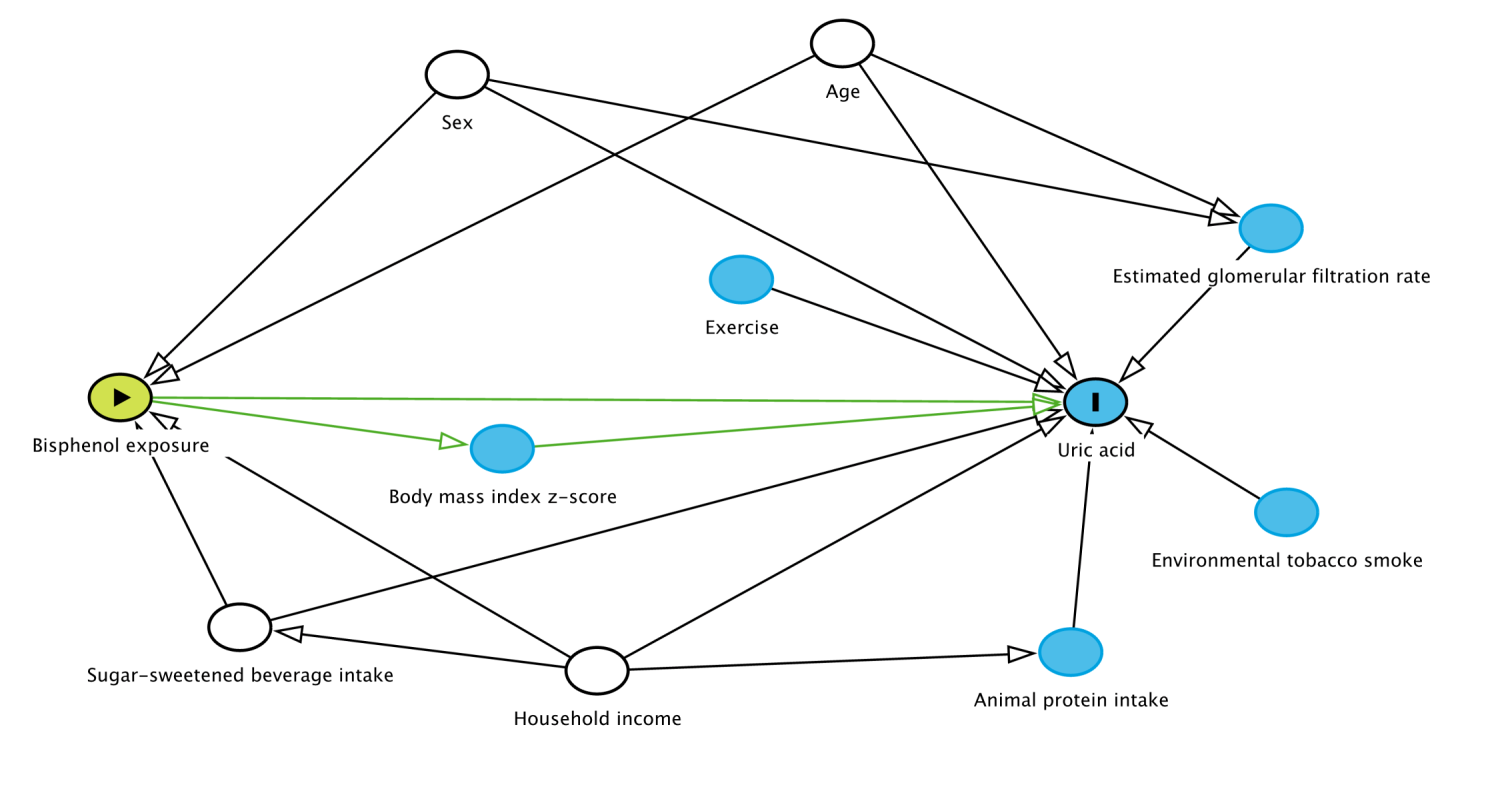

Supplement: S1 Fig — Directed acyclic graphs (DAGs) show the hypothesized causal relationship between bisphenol exposure (yellow circle), covariates, and serum uric acid concentrations (‘I’ in a blue circle). The proposed adjustment variables are indicated by white circles and the proposed adjustment variables in the model are indicated by blue circles. (TIF) [file pone.0268503.s009.tif]
